# A Grand Challenge for Personality and Social Psychology: Competition, Cooperation, or Co-existence?

**DOI:** 10.3389/fpsyg.2020.01570

**Published:** 2020-07-23

**Authors:** Gerald Matthews

**Affiliations:** Institute for Simulation and Training, University of Central Florida, Orlando, FL, United States

**Keywords:** personality, social psychology, methodological pluralism, biopsychology, social processes

## How Can We All Get Along?

Like two rival siblings, the disciplines of personality and social psychology have common roots but an evolving and sometimes difficult relationship (Pettigrew and Cherry, 2012; Lanning, 2017). Both are diverse and have their own internal controversies, but, historically, the field has been divided according to two worldviews (Cloninger, 2020). Personality trait researchers favor a natural sciences approach, characterized by a search for general, nomothetic principles for understanding relationships between quantitative individual-difference variables (Boyle et al., 2008). They are sympathetic to biological explanations for trait variation, expressed in studies of evolutionary bases, behavior and molecular genetics, and neuroscience. By contrast, social constructivists are attuned to qualitative, idiographic studies of the ways in which people interact within a specific sociocultural milieu, with personality negotiated dynamically “between” as much as “within” people (Hampson, 1988). They also favor a humanistic over a natural-sciences orientation, which values efforts by psychologists to support individual flourishing and social justice (Cloninger, 2020).

Neither worldview is monolithic. For example, on the trait side, variation in traits associated with the self has been attributed to motivational and cognitive factors rather than direct neurological influences (Ryan and Deci, 2017). Experimental social psychology lends itself to nomothetic theories, such as those focused on social cognition. Nevertheless, the tension between natural-science and humanistic perspectives (Cloninger, 2020) threatens the unity and integrity of the field.

How can different networks of researchers get along with one another? There have been periods of direct *competition*: famously, Mischel's (1968) “situationist” critique of personality traits. Here, the clash of ideas was productive in leading to high-quality studies of cross-situational consistency that supported an interactionist model of personality (Funder, 2006). However, competition also risks generating empty rhetoric without scientific progress. In fact, much of the time, trait researchers and social psychologists *co-exist* but largely ignore one another's work, potentially missing opportunities for exchange of ideas. Happily, recent years have seen increased *cooperation* and theories that integrate the two fields. Often, such efforts require unpacking of the logically distinct elements of each perspective. For example, social-cognitive theory points toward psychological attributes, such as the self-schema that can be stable over time without being a direct expression of brain functioning.

The grand challenge for personality and social psychology is to define the research problems for which competition, co-existence, and cooperation are appropriate. In this article, I emphasize prospects for cooperation: personality and social psychologists have much to learn from one another. However, while debate should always be collegial, cooperation may not be the answer for some topics. For certain questions one camp may have better evidence-based answers than the other. Other topics may only be amenable to investigation from one perspective, calling for civil co-existence without cooperation or competition.

## What Personality Trait Psychologists Can Learn From Social Psychology

A paradox of traditional personality trait research is that individual differences in social behavior are central to many traits, notably extraversion, and agreeableness, but theory has had relatively little to say about underlying social processes. More recent work has remedied this gap and the importance of integrating trait and social-psychological theory is better recognized. Some examples follow:

### Social Processes Are Important for Personality Development

Behavior-genetic studies indicate that at least half of the variance in major traits is attributable to the environment, primarily “non-shared” influences operating at the level of the individual rather than the family (Plomin, 2011; Turkheimer et al., 2014). Several research directions help to fill the gaps in understanding of personality development. The idea that the self is socially constructed is familiar to social psychologists. From a personality perspective, individual differences in the acquisition of self-regulative capabilities in childhood influence trait development guided by socially endorsed reference values or norms (Denissen et al., 2013). Over the lifespan, the neo-socioanalytic theory of Roberts and Nickel (2017) emphasizes investing in social institutions as a driving mechanism for personality development, supported by evidence for plasticity of traits in adulthood. The studies cited illustrate how an adequate account of trait development requires specification of how biological and social factors interact dynamically; it is not enough to assume that the genotype feeds forward directly into the individual's trait composition (Matthews, 2016).

### Culture Affects the Dimensional Structure of Traits

A pillar of biological trait theory is the claim that major traits, such as the Big Five are universal across cultures (Costa and McCrae, 1992). However, while dimensions resembling the Big Five are indeed found in most studies, data often fail to meet criteria for true measurement invariance, especially in non-Western and non-industrialized cultures (Church, 2016). Personality research also identifies culture-specific “emic” or indigenous traits. There is a clear opportunity for personality researchers to examine the social processes that shape cultural differences, including the impact of cultural norms for beliefs, values, and interpersonal communication (Lehman et al., 2004). Understanding of group and individual differences in such processes may in turn lead to insights into the role of gene-culture interactions in personality (Church, 2016).

### Social Context Matters

Interactionism has always been central to personality research but traditional biological theories admit only a narrow range of situational factors, such as presence and intensity of positive and negative reinforcers (Boyle et al., 2008). Within trait personality itself, this view is challenged by studies showing that context specific-measures, such as those for work self-efficacy and evaluative anxieties are often more predictive in-context than general measures (Matthews, 2020b). Within social psychology, a major contribution is the identification of “behavioral signatures” as elements of personality consistency (Mischel and Shoda, 1995), i.e., if-then relationships that specify the individual's typical cognitive, affective and behavioral reactions to specific social contexts. Accounts of narrative identity provide another way to understand stability of personality from a social perspective (McAdams and McLean, 2013). Thus, the social-psychological perspective enhances the capacity of trait models to handle situational factors.

### Social Processes Mediate Personality Effects

Following from the previous point, social-psychological theory enriches understanding of the influence of personality traits on subjective experience and behavior. Social-cognitive theories of self-regulation identify processes, such as self-verification and self-presentation that vary systematically across individuals and can mediate trait effects (Robinson and Sedikides, 2020). Understanding traits for negativity, such as neuroticism and trait anxiety is a case in point. While basic brain mechanisms, such as sensitivity to punishment play a role, individuals high in these traits often display specifically social vulnerabilities and characteristic core self-evaluations (Chang et al., 2012). Process-oriented work on relevant traits, such as rejection sensitivity (Berenson et al., 2009) contributes to understanding the social expression of major traits. More generally, social-cognitive theories provide a wealth of dynamic process models that can be integrated with trait accounts of personality (Cervone, 2005).

## What Social Psychologists Can Learn From Trait Psychology

Stable individual differences in social behavior are now well-established. So too is the role of biology in social behavior (Cacioppo et al., 2010). This does not mean that every social-psychological research question must be addressed from a biological standpoint, but there are opportunities for social psychology to develop multileveled theories that include biological factors.

### Social Psychological Constructs Can Be Measured and Validated According to Contemporary Test Standards

Traditionally, social psychologists are skeptical about measurement of broad individual dispositions divorced from social context. Nevertheless, numerous scales have been developed for core social-psychological constructs including values, concerns with social evaluation, intergroup contact, stereotyping, and prejudice, attitudes toward sexual orientation, and others (Boyle et al., 2015). Such scales are validated against the same criteria used for personality and other individual-difference measures. Obviously, measuring prejudice with a self-report scale is insufficient for understanding the construct, but the scale provides a tool for capturing some (explicit) elements of prejudice and investigating how personal dispositions interact with situational factors.

### Traits Influence Social Behaviors

Just as trait psychologists should recognize the importance of social processes, so too should social psychologists recognize systematic, dispositional influences on social behavior. These influences are captured by facet-level traits, such as social anxiety, rejection sensitivity, sociability, self-consciousness, and many others. The interpersonal circumplex provides a broad framework for understanding social traits that can be mapped to trait dimensions (Gurtman, 2009). Traits are important too for understanding personality disorders that negatively impact social functioning. For example, it would be difficult to fully explain criminal and antisocial behavior without reference to the Dark Triad (or Tetrad) (Paulhus, 2014). Some social psychologists may harbor skepticism about the value of trait accounts from a reasonable desire to avoid crude biological reductionism. However, understanding of abnormal social psychology benefits from an integration of perspectives.

### Traits Influence Values and Political Attitudes

The relationship between personality and moral character is a theme going back to antiquity. Various instruments measuring moral dispositions and values (Campbell et al., 2015) can inform relevant social-psychological studies. Similarly, the rise of populism has revived interest in individual differences in political attitudes and their underpinnings including right-wing authoritarianism, nationalism, and ethnocentrism. Evidence linking conservatism to brain systems for threat processing suggests a biological component to individual differences (Pedersen et al., 2018).

### Development Confounds Social Factors With Biology

The influence of biologically-based temperamental factors on child development (Rothbart et al., 2020) has profound implications for understanding social factors. Environmental and genetic factors may be correlated. Suppose we find that troubled parents tend to have troubled children. Is this because the parents adopt a suboptimal parenting style, or because parents and children share genes for personality? More nuanced accounts of gene-environment correlation and interaction suggest multiple developmental processes (Rutter, 2012; Cicchetti, 2016). The parents' genes shape their behaviors toward the child, and hence the environment in which it grows up. The child's genes influence the environments it selects for itself, and the behaviors it elicits from parents and others (Rutter, 2012). Maladaptive environments interact with genes to affect brain development (Cicchetti, 2016). Such interactions include epigenetic effects, where environment influences gene expression (Rutter, 2012). These complex and subtle effects do not negate the value of social-cognitive perspectives on child development, but they do imply that a full account of individual differences in social learning must accommodate biological factors. The advent of social neuroscience (Cacioppo et al., 2010) provides paths toward mechanistic explanations. As these authors discuss, social behavior may reflect reciprocal interactions between social and biological antecedents.

## Limits to Cooperation

Unity is typically valued as a goal for personality and social psychology. For example, a recent Special Issue of *Personality and Individual Differences* (Corr, 2020) addresses the question of what a consensual paradigm for personality would like. The mutual benefits of dialogue between personality and social psychology suggest unity is feasible as well as desirable. However, there are also reasons for continued separation of some fields of inquiry. Unity implies reductionism, e.g., group-level constructs to individual-level constructs, cognitive processes to neural processes, and neural processes to gene expression. Because higher-level constructs can emerge from lower-level ones in complex ways, reductionism has its limits as a basis for the field. Instead, we should seek explanatory pluralism, i.e., multiple levels of explanation whose constructs do not necessarily map onto one another (Matthews, 2020a). Some research can explore integration of levels for specific problems (Cacioppo et al., 2010); in other cases, no integration may be possible, and the goal is to choose the single level that is most appropriate. Next, I give two examples of problems where integration across levels appears problematic.

### Group-Level Analyses

Multi-leveled organizational theory identifies levels of analysis that characterize groups. Kozlowski and Klein (2000) differentiated three categories of team-level constructs: shared, configurable and global constructs. Shared constructs mirror individual-level ones; for example, team self-efficacy may be a composition of individuals' self-efficacy. Configural constructs, such as team diversity, are emergent from individual characteristics, but are conceptually distinct from them. Global constructs refer to the team as a unit, such as its function at work, with no reference to individuals. Studies addressing configural and, especially, global constructs are unmoored from personality and individual differences. Furthermore, group-level constructs can be defined at different levels themselves, ranging from teams to organizations. In certain contexts, it is thus legitimate for social psychologists to work at the group-level only.

### Limits of Genetics

Does genetic modeling of personality variance actually tell us anything about causal processes? Turkheimer et al. (2014) argue that while personality traits universally show heritabilities of ~0.4, such findings tell us little about behavioral expressions of the trait phenotype. Individual genes have vanishingly small effects on personality, and so even molecular genetic studies do not lead to specifications of specific biological mechanisms. Behavior genetics provides insight into correlational data, such as associations between parent and child personalities, but it is irrelevant to causal hypotheses. Not all geneticists would agree, but according to Turkheimer et al. (2014, p. 535), “Personality is heritable, but it has no genetic mechanism.” Similar cautions have been expressed about the inferences to be drawn from genome-wide association studies (GWAS: Feldman and Ramachandran, 2018).

## Conclusion

Traditional perspectives on personality and on social psychology have more common ground than is sometimes appreciated. Happily, the two fields are coming closer together, but cooperation could be much enhanced. Developments in social cognition and social neuroscience provide a common scientific language in support of integration. At the same time, not all research can be or should be cooperative. The flip side of cooperation is competition. Researchers with different scientific worldviews may have different hypotheses for phenomena. Commonality of constructs allows hypotheses to be tested against one another. There may be winners and loser, or, alternatively, a synthesis of contrary views. In other cases, there may be no grounds for dialogue between researchers with different perspectives, and the research topic belongs to one or other discipline. Group-level processes and genetic factors are possible examples. Such cases call only for co-existence, though it remains open to researchers to argue otherwise. In addition, practical efforts to tackle social issues may require fine-grained contextualized understanding that does not mesh well with more general theories. The over-arching challenge is to establish which research questions and practical goals are open to integration of different viewpoints, and which are not. The Personality and Social Psychology section continues to welcome contributions across the spectrum of research in this field, but I especially encourage submissions that engage with multiple perspectives.

## Author Contributions

The author confirms being the sole contributor of this work and has approved it for publication.

## Conflict of Interest

The author declares that the research was conducted in the absence of any commercial or financial relationships that could be construed as a potential conflict of interest.
